# Exercise and physical performance in older adults with sarcopenic obesity: A systematic review

**DOI:** 10.3389/fendo.2022.913953

**Published:** 2022-07-28

**Authors:** Laura Ghiotto, Valentina Muollo, Toni Tatangelo, Federico Schena, Andrea P. Rossi

**Affiliations:** ^1^ Department of Neuroscience, Biomedicine and Movement, University of Verona, Verona, Italy; ^2^ Department of Medicine, University of Verona, Verona, Italy; ^3^ Geriatrics Division, Department of Medicine, Ospedale Cà Foncello ULSS2, Treviso, Italy; ^4^ Healthy Aging Center, Department of Medicine, Division of Geriatrics, University of Verona, Verona, Italy

**Keywords:** physical performance, physical function, muscle mass, exercise, elderly, sarcopenic obesity, systematic literature review

## Abstract

**Background:**

Sarcopenic obesity is characterized by low muscle mass and high body fat; prevalence increases with age, particularly after age 65 years. For this systematic literature review we searched scientific databases for studies on exercise interventions for improving physical performance in adults with sarcopenic obesity; also, we identified potential gaps in clinical practice guidelines that need to be addressed.

**Methods:**

We followed the recommendations of the Preferred Reporting Items for Systematic Reviews and Meta-Analyses (PRISMA). The databases were searched for studies published through November 2021 that measured physical performance in adults with sarcopenic obesity.

**Results:**

Most of the studies applied a strength training protocol in which improvement was noted post-treatment on the Time Chair Rise (TCR), 30-s Chair Stand, and Single Leg Stance (SLS) tests. Discrepancies between the studies were observed when resistance training was combined with or without elastic bands or electromyostimulation, as measured with the Short Physical Performance Battery (SPPB), Physical Performance Test (PPT), Gait Speed, and Timed Up & Go (TUG) test. Post-intervention SPPB, PPT, and gait speed scores showed an increase or maintenance of performance, while TUG test scores were higher according to one study but lower according to another.

**Conclusions:**

Engagement in physical exercise, and resistance training in particular, can improve or maintain physical performance in adults with sarcopenic obesity. Study samples should include more men. A future area of focus should be the impact of different types of training (aerobic, power training, combined modalities). Finally, studies with longer intervention periods and follow-up periods are needed to gain a better understanding of the effectiveness of exercise on physical function in adults with sarcopenic obesity.

## Introduction

One of the major public health challenges of the 21st century is obesity. In simple terms, obesity is an excessive increase in fat mass (1). Since the 1980s, its prevalence has tripled in many European countries and the population with weight excess continues to grow at an alarming rate (2). Obesity impacts on morbidity, disability, activities of daily living and increases the risk of developing cardiovascular disease (e.g., heart disease and stroke), type 2 diabetes, musculoskeletal disorders (osteoarthritis), some cancers, and other health-related issues (3).

Musculoskeletal disorders in the aging population are a growing public health concern (4). A prominent change associated with human aging is the progressive decline in skeletal muscle mass and strength. Several studies have suggested that muscle mass declines by nearly 6% per decade after mid-life (4). The percentage of loss of muscle strength per year is 50 to 100% greater than the loss of muscle mass. In the population of the Health ABC Study, the annual rate of decline in leg strength was approximately three times greater than the rate of loss of lean leg mass (~1% per year) (5).

Sarcopenia, derived from the Greek sarx (flesh) and penia (loss), was introduced by I.H. Rosenberg in 1989 (6) to describe the loss of skeletal muscle mass. The term refers to an age-related, progressive, generalized skeletal muscle disorder (7) associated with physical disability, metabolic dysfunction, and increased mortality (8).

Research groups in Europe and Asia have developed consensus on the definition and diagnostic criteria for sarcopenia. In 2010, the European Working Group on Sarcopenia in Older People EWGSOP (9) defined sarcopenia as the presence of both low muscle mass and low muscle strength or performance. In 2019, the EWGSOP updated the definition of sarcopenia diagnosis (EWGSOP2). In its revised definition, the EWGSOP2 recommends the use of low muscle strength (evaluated with the handgrip strength or the chair stand test) as the primary parameter for screening, subsequently confirmed by low appendicular skeletal muscle mass adjusted by height in meters squared.

Two other research groups, the International Working Group on Sarcopenia (IWGS) (10) and the Asian Working Group for Sarcopenia (AWGS) (11) have adopted similar approaches to defining sarcopenia as the presence of low appendicular skeletal muscle mass and poor muscle function.

A related disorder is sarcopenic obesity, a term introduced by Baumgartner (12) in reference to the co-presence of sarcopenia and obesity in a specific phenotype of low muscle mass and high body fat. As the population ages, the prevalence of sarcopenic obesity increases, as the prevalence of obesity and sarcopenia also increases, particularly among adults aged 65 years or older (13). It is associated with a reduction in physical activity and energy expenditure and an increase in body weight. Sarcopenia and obesity result in reduced physical performance (14). A hallmark of sarcopenia is slower gait speed. Besides the higher risk of falls (15), older people with obesity have reduced physical function, as assessed *via* self-assessment questionnaires or tests such as the Short Physical Performance Battery (SPPB) (14). Sarcopenic obesity is thought to have a synergistic effect on health deterioration compared to sarcopenia or obesity alone. It is responsible for more health problems than either sarcopenia or obesity (16) and is a leading cause of metabolic disorders, disability, cardiovascular disease (4), and mortality. A shared definition is currently lacking, making it difficult to establish standardized diagnosis and management. While progress has been made in defining sarcopenic obesity according to the recent Consensus of the European Society for Clinical Nutrition and Metabolism (ESPEN) and the European Association for the Study of Obesity (EASO) (17), on treatment of the condition the discussion is still open.

Prevention and treatment hold importance for public health and individual healthy aging. Exercise strategies have been developed to improve cardiovascular and metabolic function (18), cancers survival (19), and to increase muscle mass, muscle strength, and physical performance in adults with sarcopenic obesity (20). The mechanisms by which physical exercise can induce beneficial effects in sarcopenia and obesity are multifactorial. For example, exercise plays an essential role in regulating the energy balance which, when combined with a low-calorie diet, can set a lower energy balance. Also, exercise can enhance physical functioning parameters, such as handgrip strength, gait speed, and balance capacity in adults with sarcopenia and those with obesity (21). Notably, improvement is closely linked to exercise intensity, volume, frequency, and workout progression. Since exercise is an effective strategy for improving body composition in individuals with sarcopenia and those with obesity, regular exercise can play a central role in treating sarcopenic obesity (8, 18).

Resistance exercise is recognized as an effective strategy for increasing muscle hypertrophy and improving muscle function and strength in older adults (22). Most studies involve healthy older populations, while some reviews or meta-analyses evaluate studies on exercise in combination with amino acid or protein supplementation (18). Limitations that explain the low impact of exercise interventions include lack of standardization of exercise protocols, short duration of interventions, and differences in eligibility criteria (23).

For this systematic review we searched the scientific literature on types of exercise designed to improve physical performance in adults with sarcopenic obesity; also, we identified and analyzed potential gaps in clinical practice guidelines that merit attention in future studies.

## Methods

This systematic review was conducted according to the Preferred Reporting Items for Systematic Reviews and Meta-analyses (PRISMA) 2020 statement and our review methods were established prior to data extraction and were pre-registered with PROSPERO (ID: CRD42022314354).

### Identification guidelines

One researcher (LG) carried out the literature search to identify studies of exercise treatment and physical performance in older adults with sarcopenic obesity. The PubMed, Scopus, EBSCO and Cochrane Library databases were searched up to November 2021, without language or publication date restrictions.

### Search terms

The search was performed using the keywords: OLDER ADULTS: “elder” OR “elderly” OR “elders” OR “aged” OR “seniors” OR “senior” OR “older” OR “old people” OR “older people” OR “aging”. SARCOPENIC OBESITY: “sarcopenic obese” OR “sarcopenic obesity” OR “sarcopenia obese” OR “sarcopenia obesity” OR “obese sarcopenic” OR “obesity sarcopenic” OR “obese sarcopenia” OR “obesity sarcopenia”. EXERCISE: “training” OR “exercise” OR “resistance training” OR “strength training” OR “resistance exercise” OR “strength exercise” OR “aerobic training” OR “aerobic exercise” OR “high speed circuit training” OR “power training”. PHYSICAL TEST: “short physical performance battery” OR “physical performance test” OR “gait speed” OR “walking speed” OR “chair stand” OR “time chair rise” OR “single leg-stance” OR “one leg balance” OR “time up and go”.

### Inclusion and exclusion criteria

- Original research findings (reviews, meta-analyses, editorials, conference abstracts, research protocols were excluded)
- Observational and experimental studies (reference data only, abstracts excluded if no data could be extracted)
- Study sample involving women and men of any race, age ≥60 years with a detailed diagnosis of sarcopenic obesity, ability to undertake bipedal locomotion
- All exercise interventions included in the analysis

Non-human studies and studies not reported pre-post intervention change in outcome (e.g., cross-sectional studies) were excluded.

### Data extraction

The records were processed using Rayyan-Intelligent Systematic Review software, which detected duplicates (Systematic Reviews (2016) 5: 210, DOI: 10.1186/s13643-016-0384-4.). Each duplicate was manually checked before removal. The records were identified and then screened; the abstracts were reviewed, and the full text then analyzed when the abstracts were unclear. Finally, the records were selected for analysis.

Two authors (LG; TT) independently extracted the data from the studies and entered them into a Microsoft Excel spreadsheet; disagreement was resolved by consensus. **Table 1** presents a summary of the study characteristics: 1) authors, 2) publication year, 3) country, 4) sample size and characteristics of the study population, 5) sex of the study population, 6) age of the population, and 7) study design. One author extracted the data, and another checked the extracted data. **Table 2** presents the characteristics of the studies 1) definition of sarcopenic obesity, 2) types of intervention and physical performance tests, 3) aim of the study and outcome for physical performance, 4) duration of the intervention, and 5) outcome for physical function.

**Table 1:** General characteristics of the studies.

Author	Year	Country	Sample size	Groups	Sex	Age (m ± SD)	Study design
Balachandran	2014	USA	17, M/W	1) SH 2) HSC	1) M: 1, W: 8 2) M: 0, W: 8	1) 71.0 ± 8.2 2) 71.6 ± 7.8	RANDOMIZED CONTROLLED TRIAL
Vasconcelos	2016	SPAIN	31, W	1) EX 2) C	1) 16 2) 15	1) 72.0 ± 4.6 2) 72.0 ± 3.6	RANDOMIZED CONTROLLED TRIAL
Kim	2016	JAPAN	139, W	1) EX+N 2) EX 3) N 4) C	1) 36 2) 35 3) 34 4) 34	1) 80.9 ± 4.2 2) 81.4 ± 4.3 3) 81.2 ± 4.9 4) 81.1 ± 5.1	RANDOMIZED CONTROLLED TRIAL
Kemmler	2016	GERMANY	75, W	1) WB-EMS 2) WB-EMS + PS 3) C	1) 25 2) 25 3) 25	1) 77.3 ± 4.9 2) 76.4 ± 2.9 3) 77.4 ± 4.9	RANDOMIZED CONTROLLED TRIAL
Liao	2017	TAIWAN	46, W	1) RT 2) C	1) 25 2) 21	1) 66.4 ± 4.5 2) 68.4 ± 5.9	CLINICAL TRIAL
Stoever	2018	GERMANY	48, M/W	1) SAR 2) NSAR	1) M: 20, W: 5 2) M: 16, W:14	1) SAR: M: 71.0 ± 4.3 W: 72.2 ± 5.4 2) NSAR: M: 69.6 ± 3.7 W: 68.2 ± 2.2	RESEARCH REPORT
De Oliveira Silva	2018	BRAZIL	49, W	1) SO + RT 2) RT	1) 8 2) 41	1) 66.9 ± 3.3 2) 66.0 ± 4.0	ORIGINAL RESEARCH
Liao	2018	TAIWAN	50, W	1) RT 2) C	1) 30 2) 20	1) 66.7 ± 4.5 2) 68.3 ± 6.0	RANDOMIZED CONTROLLED TRIAL

C, control; EX, exercise; HSC, high speed circuit; M, men; m ± SD, mean ± standard deviations; NSAR, obese +/- pre-sarcopenia; PS, protein supplementation; RCT, randomized controlled trial; RT, resistance training; SH, strength / hypertrophy; SO, sarcopenic obesity; W, women; WB-EMS, whole-body electromyostimulation.

**Table 2:** Characteristics of the studies.

Author	Year	Definition of Sarcopenic Obesity		Assessment Tool of Body Composition	Type of exercise intervention	Type of physical performance test	Aim	Outcome	Time Point of Measurement	Results
		Obesity	Sarcopenia							
Balachandran	2014	BMI >30 kg/m^2^	SMI (TSM/Ht^2^) <10.76 kg/m^2^ M, <6.76 W or HG <30 M, <20 W kg or GS <1m/s	BIA	SH versus HSC	SPPB	Compare the effects between HSC and conventional SH training on neuromuscular performance, body composition and IADL function	SPPB	Baseline: 0 weeks Posttest: 15 weeks	SPPB: HSC ↑ 20%; SH ⇌
Vasconcelos	2016	BMI ≥30 kg/m^2^	HG ≤21 kg	NA	RET	SPPB, 10-m walk test	Evaluate the effects of a progressive RET program with high-speed component on the physical function	SPPB, 10-m walk test	Baseline: 0 weeks Posttest: 10 weeks	SPPB, GS ⇌
Kim	2016	%BF ≥32%	SMI (ASM/Ht^2^) <5.67 kg/m^2^ or HG <17 kg or GS <1 m/s	BIA	chair exercise *vs* resistance band exercise *vs* hydraulic exercise machine *vs* aerobic training	5-m walk test	Investigate the effects of exercise and/or nutritional supplementation on body composition, blood components, and physical function in community-dwelling elderly Japanese	Physical function (5-m walk test)	Baseline: 0 weeks Posttest: 12 weeks	GS ⇌
Kemmler	2016	%BF >35%	SMI (ASM/Ht^2^) <5.75 kg/m^2^	DXA	WB-EMS	10-m gait speed	Determine the effect of WB-EMS in community-dwelling women	GS	Baseline: 0 weeks Posttest: 26 weeks	GS ↑
Liao	2017	%BF >30%	SMI (TSM/Ht^2^) <7.15 kg/m^2^	BIA	ERET	SLS, 10-m GS, TUG, TCR	Identify the clinical efficacy of RET	Physical capacity (SLS, GS, TUG, TCR)	Baseline: 0 weeks Posttest: 12 weeks	SLS, TUG, TCR, GS ↑
Stoever	2018	BMI ≥30 kg/m^2^	SMI (%) ≤37% M, ≤27.6 W or HG ≤32 M, ≤21 W kg or GS <0.8m/s	BIA	RET	SPPB, PPT	Investigate the influence of resistance training on physical function	Physical function (SPPB)	Baseline: 0 weeks Posttest: 16 weeks	SPPB, PPT ↑
De Oliveira Silva	2018	BMI >27 kg/m^2^	AFFM DEXA - predicted AFFM = ≤3.4	DXA	RET	30-second chair stand test, TUG	Compare the effects of RET on body composition, muscle strength, and functional capacity	Physical function (Chair stand, TUG)	Baseline: 0 weeks Posttest: 16 weeks	Chair stand ↑ TUG ↓
Liao	2018	BMI >30 kg/m^2^	SMI (TSM/BW) <27.6%	BIA	ERET	SLS, 10-m GS, TUG, TCR	Identify the effect of ERET on muscle mass and physical function	Physical capacity (SLS, GS, TUG, TCR)	Baseline: 0 weeks Posttest: 12 weeks	SLS, TUG, TCR, GS ↑

AFFM, Appendicular fat-free mass; ASM, appendicular skeletal muscle mass; BIA, bioimpedance analysis; BMI, body-mass index; BW, body weight; DXA, dual X-ray absorptiometry; ERET, elastic resistance exercise training; GS, gait speed; HG, handgrip; HSC, high-speed circuit; Ht, body height; NA, the information was not given in the manuscript; PPT, Physical Performance Test; RET, resistance exercise training; SH, strength/hypertrophy; SLS, Single-leg stance; SMI, skeletal muscle index; SO, sarcopenic obesity; SPPB, short physical performance battery; TCR, Timed Chair Rise; TSM, total skeletal muscle; TUG, time up and go; VFA, visceral fat area; WB-EMS, whole-body electromyostimulation; %BF, body fat percentage; ⇌, no change; ↑, improved; ↓, decreased.

### Quality assessment and Risk of Bias

**Figure 1:**
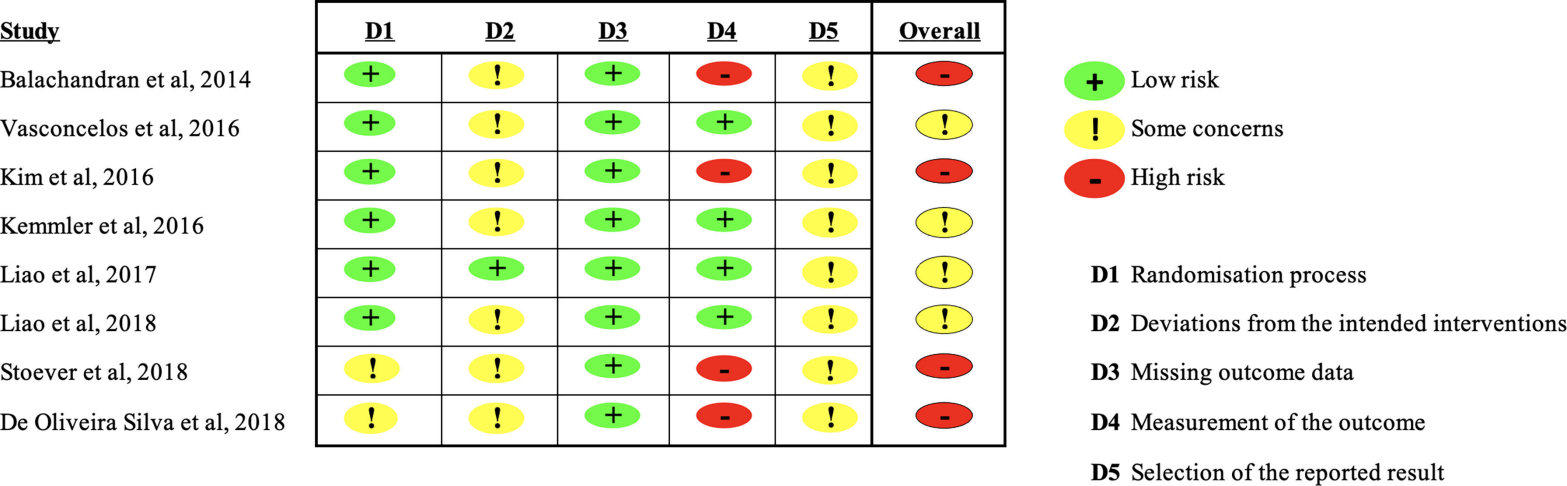
Summary of Risk of bias in the included studies.

Two authors independently assessed the methodological quality of the studies using the Risk of Bias Assessment Tool for Randomized trials (RoB2) (24) (**Figure 1**). Study quality was rated high, moderate, or low based on study design and risk of bias. Two authors (LG and TT) independently evaluated the studies; disagreement was resolved by discussion and consensus. A third reviewer (VM) was consulted if needed.

## Results

### Study selection

**Figure 2:**
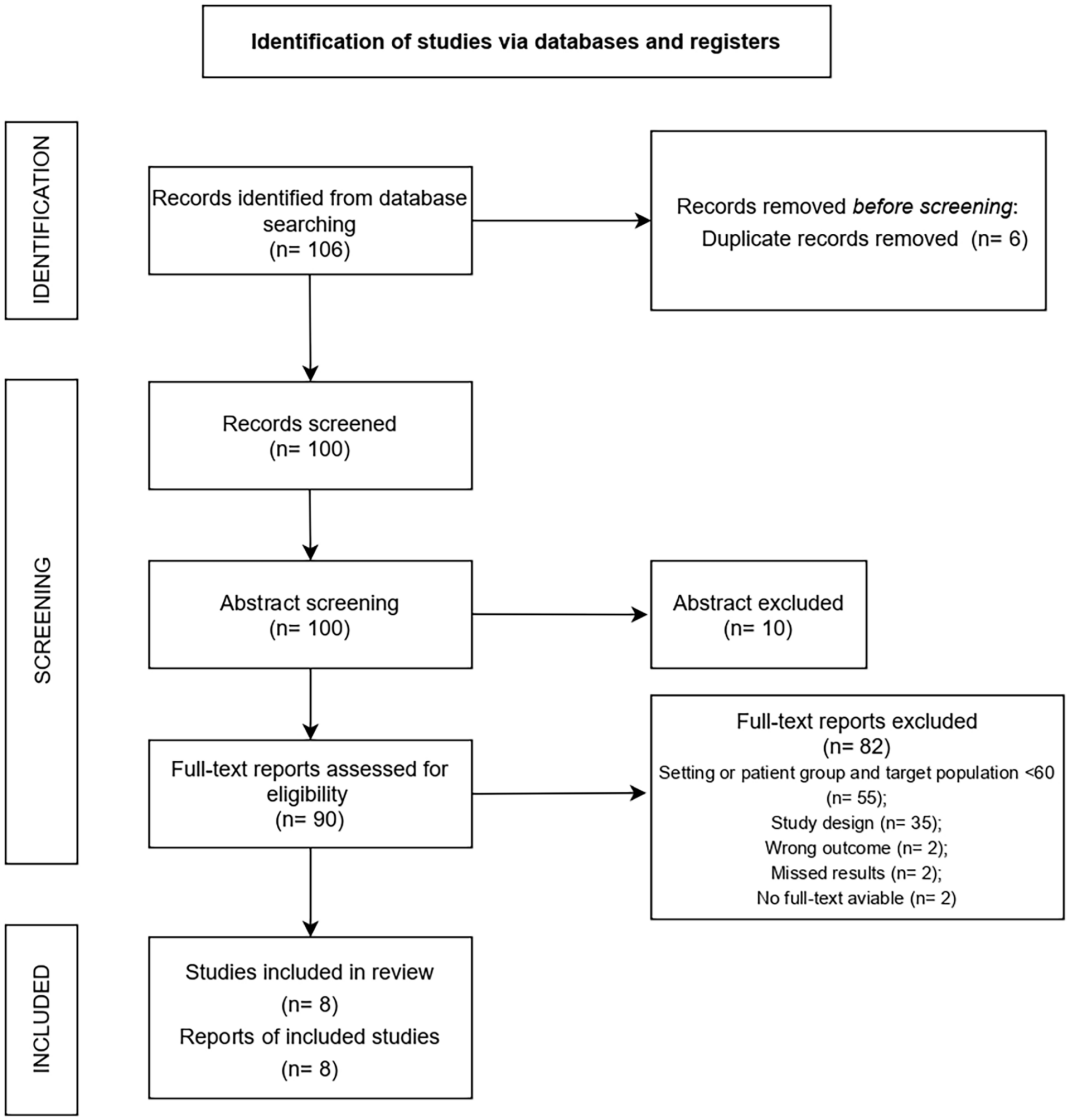
Flow chart.

A total of 106 studies were retrieved. After removing duplicates, 100 studies were screened based on title and abstract. After screening, 90 full texts were evaluated. Records were excluded because: study population not conform with inclusion criteria (n=55), study design not conform with inclusion criteria (n=35), outcome did not meet inclusion criteria (n=2), no results were reported (n=2), no full text was available (n=2). The final analysis included 8 studies, 6 of which were randomized controlled trials (RCT) (25–30), 1 was a research report (31), and 1 was an original research article (32) (**Figure 2**).

### Study characteristics

The study population was composed of women in 6 studies (26–30, 32) and of both men and women in 2 (25, 31). Participant age ranged from 60 to 80 years. In detail, the interventions involved resistance training (25, 26, 29, 30, 32) in the patient samples aged from 68 to 72 years (**Table 1**). While other types of exercise, such as chair exercise *vs* resistance band exercise *vs* hydraulic exercise machine *vs* aerobic training intervention (27) or muscle stimulation (28), where used in samples between 77 and 81 years of age. Other kinds of exercises were not employed (**Table 1**).

### Type of interventions

Obesity was categorised by body-mass index (BMI, weight in kg divided by height in meters squared) in 5 studies (cut off 27 kg/m^2^ in (32) and 30 kg/m^2^ in (25, 26, 30, 31)) and by body fat percentage (%BF) in 3 studies (cut off point 30% in (29), 32% in (27) and 35% in (28)). Sarcopenia was defined according to skeletal muscle mass (total skeletal mass (TSM) divided by body height in meters squared (Ht^2^), appendicular skeletal mass (ASM)/(Ht^2^), skeletal muscle index percentage (SMI%), ideal appendicular fat-free mass (AFFM) or TSM/BW in (25, 27–32), and handgrip strength in (25–27, 31) (**Table 2**).

Physical performance was measured with the SPPB in (25, 26, 31) and with the Physical Performance Test (PPT) in (31), the walk test (10-m walk test or 5-m walk test or 10-m GS) in (26–30), the Single Leg-Stance (SLS) in (29, 30), and the Time Up & Go test (TUG) or the Timed chair rise (TCR) or the 30-s chair stand test in (29, 30, 32) (**Table 2**).

### Effects of exercise on physical performance

Six studies investigated the effects of resistance exercise on physical performance (26, 27, 29–32); one evaluated the effects of strength exercise *vs* high-speed circuit on physical performance (25), and one with electromyostimulation (28) (**Table 2**). Overall, there was a statistically significant increase in physical performance scores on the SPPB, PPT, SLS, TUG, TCR, GS, and 30-s chair stand (29–32) after resistance exercise intervention One study reported a statistically significant decreases in TUG scores (32) and 2 studies found no statistically pre/post change in SPPB and 10-m GS (26, 27). One study (25) comparing the effects of a strength exercise *vs* a high-speed circuit noted an increase in SPPB scores for the high-speed circuit group but no change in scores for strength/hypertrophy group. The one study (28) that used electromyostimulation found a post-treatment increase in the 10-m GS (**Table 2**).

### FITT table and adherence to the intervention

**Table 3** presents the training protocols following the FITT principle: frequency, intensity, time, and type of training. Furthermore, adherence was added since it is a key component in exercise interventions (33–36). Training frequency differed between studies: 26 weeks of training in (28), and 10-16 weeks in (25–27, 29–32). Exercise intensity during training sessions also varied between studies: one repetition maximum (1RM) in (25, 26, 31, 32), rate of perceive exertion (RPE) in (28–30), and a combination of exercise and weight progression in (27) although the method was not specified. The duration of training sessions ranged between 60 min/day in (25–27, 31) and 15 to 45 minutes in (28–30, 32). Five studies (25, 26, 28–30) reported high adherence (≥81%), while the others 3 studies did not report adherence rates.

**Table 3:** Training protocol according to the FITT scheme.

Author	Year	Frequency	Intensity	Time	Type	Adherence
Balachandran	2014	2 day/w, 15 weeks	SH: 70% 1RM, increases 5%	*SH*: 55-60 min/day, 3 sets of 10-12 reps, (2s for both concentric and eccentric phase), 1-2 min rest	SH *vs* HSC	SH: 85.0% HSC: 81.0%
			HSC: 50-80% 1RM, increases 5%	*HSC*: 40-45 min/day, 3 sets of 10-12 reps, (11 machine without rest, upper and lower exercises, fast concentric phase, eccentric phase in 2s), 1-2 min of rest after each circuit		
Vasconcelos	2016	2 day/w, 10 weeks		60 min/day. Exercise only for the lower limb strength. Rest of 30s between sets and 60s between exercise	RET	85.0%
			50% 1RM	**Week 1-2**: 2 sets of 8-12 reps (concentric and eccentric phase low speeds)		
			75% 1RM	**Week 3-4**: 2 sets of 8-12 reps (concentric and eccentric phase low speeds)		
			40% 1RM	**Week 5-6**: 2 sets of 8-12 reps (concentric and eccentric phase high speeds)		
			60% 1RM	**Week 7-8**: 2 sets of 8-12 reps (concentric and eccentric phase high speeds)		
			60% 1RM	**Week 9-10**: 3 sets of 8-12 reps (concentric and eccentric phase high speeds)		
Kim	2016	2 day/w, 12 weeks	Progressive sequence from seated to standing exercise, gradually increasing weights and the resistance in bicycle ergometer training.	60 min/day	chair exercise *vs* resistance band exercise *vs* hydraulic exercise machine *vs* aerobic training	NA
				*Chair exercise*: Progressive exercises, exercise for the lower body, 1 to 3 sets of 10 reps		
				*Resistance band exercise*: exercise for the upper and lower body		
				*Hydraulic exercise machine*: exercise for the lower body and core, 1 to 3 sets of 10 reps		
				*Aerobic exercise*: cycling for 12 min, including 1 min of cooldown, starting at 40 Watt. Progressive watt level increased throughout the 3 months		
Kemmler	2016	1 day/w, 26 weeks	RPE (0-10): 4-5, 85Hz	**Week 1-4**: 11 min/day (increment of 1 min/session) to 15 min/day, impulse duration of 4s and 4s impulse break, impulse-breadth 350 ms	WB-EMS	89.0%
			RPE (0-10): 5, 85Hz	**Week 5-8**: Increment 1 min/session up to 20 min/day, impulse duration of 4s and 4s impulse break, impulse-breadth 350 ms		
			RPE (0-10): 5-6, 85Hz	**Week 9-18**: Increment 1 min/session up to 20 min/day, impulse duration of 4s and 4s impulse break, impulse-breadth 350 ms		
			RPE (0-10): 5-6, 85Hz	**Week 19-26**: Increment 1 min/session up to 20 min/day, impulse duration of 6s and 4s impulse break, impulse-breadth 350 ms		
Liao	2017	3 day/w, 12 weeks	RPE (6-20): 10-13	35-40 min/day	ERET	97.6%
			1.32 kg at 100% elongation of the yellow elastic bands	**Week 1**: 3 sets of 10-15 reps **Week 2**: 3 sets of 15-20 reps		
			1.77 kg at 100% elongation of the red elastic bands	**Week 3**: 3 sets of 10-15 reps **Week 4**: 3 sets of 15-20 reps		
			2.27 kg at 100% elongation of the green elastic bands	**Week 5**: 3 sets of 10-15 reps **Week 6**: 3 sets of 15-20 reps		
			3.22 kg at 100% elongation of the blue elastic bands	**Week 7**: 3 sets of 10-15 reps **Week 8**: 3 sets of 15-20 reps		
			4.40 kg at 100% elongation of the black elastic bands	**Week 9**: 3 sets of 10-15 reps **Week 10**: 3 sets of 15-20 reps		
			5.99 kg at 100% elongation of the silver elastic bands	**Week 11**: 3 sets of 10-15 reps **Week 12**: 3 sets of 15-20 reps		
Stoever	2018	2 day/w, 16 weeks		60 min/day	RET	NA
			60% 1RM	**Week 1-3**: 2 sets of 12-15 reps		
			80-85% 1RM	**Week 4-16**: 3 sets of 8-12 reps		
De Oliveira Silva	2018	2 day/w, 16 weeks	3 attempts to reach 1RM with progressively heavier loads, using 3–5 minutes of rest between trials	40-45 min/day 2s concentric phase and 2s for eccentric phase	RET	NA
				**Week 1-4**: 3 sets of 12-14 RM		
				**Week 5-8**: 3 sets of 10-12 RM		
				**Week 9-12**: 3 sets of 8-10 RM		
				**Week 13-16**: 3 sets of 6-8 RM		
Liao	2018	3 day/w, 12 weeks	RPE (6-20): 10-13	40 min/day	ERET	97.6%
			1.32 kg at 100% elongation of the yellow elastic bands	**Week 1**: 3 sets of 10 reps **Week 2**: 3 sets of 20 reps		
			1.77 kg at 100% elongation of the red elastic bands	**Week 3**: 3 sets of 10 reps **Week 4**: 3 sets of 20 reps		
			2.27 kg at 100% elongation of the green elastic bands	**Week 5**: 3 sets of 10 reps **Week 6**: 3 sets of 20 reps		
			3.22 kg at 100% elongation of the blue elastic bands	**Week 7**: 3 sets of 10 reps **Week 8**: 3 sets of 20 reps		
			4.40 kg at 100% elongation of the black elastic bands	**Week 9**: 3 sets of 10 reps **Week 10**: 3 sets of 20 reps		
			5.99 kg at 100% elongation of the silver elastic bands	**Week 11**: 3 sets of 10 reps **Week 12**: 3 sets of 20 reps		

ERET, elastic resistance exercise training; HSC, high-speed circuit; NA, the related information was not given in the manuscript; RET, resistance exercise training; RM, one repetition maximum; RPE, rate of perceived exertion; SH, strength/hypertrophy.

## Discussion

This systematic review outlines the current landscape of scientific research on the impact of exercise on physical performance outcomes in older adults with sarcopenic obesity; the review also reveals several gaps that merit further investigation.

Physical activity and exercise are often used interchangeably but the terms are not synonymous. Physical activity refers to any body movement produced by skeletal muscles contraction, which then results in a substantial increase in caloric requirements compared to resting energy expenditure (37). Differently, exercise is a type of physical activity consisting of planned, structured, and repetitive body movements performed to improve and/or maintain one or more components of physical fitness (37). Exercise following American College of Sport Medicine guidelines is highly recommended for people with sarcopenic obesity (38) and is key for enhancing physical function.

The majority of the studies in this review were of heterogeneous quality and poor methodology. Despite the paucity of studies, the literature highlights that exercise, and strength training in particular, with or without elastic bands or electromyostimulation, can enhance or at least maintain physical function in older adults with sarcopenic obesity. Higher scores on physical performance have been correlated with lower risk of aging-related diseases (4). These results are corroborated by findings from a recent review (8) that underlined the importance of resistance exercise in improving physical performance in individuals with sarcopenic obesity.

In the present review, the four most common tests used to assess physical performance were SPPB (39), PPT (40), SLS (41) and TUG (42).

While there are other equally validated battery tests for people with frailty (e.g., American Alliance for Health, Physical Education, Recreation and Dance (AAPHERD) (43), Rikli and Jones test (44)), they were not examined in the literature. A possible explanations might be that the tests (SPPB, PPT, SLS,TUG) are valuable tools for standard clinical assessment in older adults because they provide fast, affordable, and reliable measures of functional capacity (45). Furthermore, these tests are among those most commonly used to assess frailty in the older population, especially in individuals with sarcopenic obesity which have a higher risk of functional disability and frailty.

### SPPB and PPT outcomes

Good physical performance mirrors the muscle capacity that older adults need to maintain independence in carrying out tasks of daily living (31). The SPPB test is often used to predict the risk of loss of independence and is a standard measure in research and clinical practice (46). Since mobility is impaired in older adults with sarcopenic obesity, one aim of this systematic review was to identify the type of exercise that could improve physical function. Previous studies (39, 47) showed that older adults with the low SPPB scores were more likely to experience disability in daily living than those with high scores. Other studies have also shown that physical exercise can enhance physical performance (48). For instance, Perera et al. (49) reported that an increase of even 1.0 point on the SPPB test signals a significant change in research, as such criteria are useful for assessing the clinical significance of an intervention. Performance measures can help to determine health and physical funtion in older adults and provide a yardstick for understanding and acting on their health needs.

Two (25, 31) of three studies that employed strength training or resistance exercise training reported post-intervention improvements in SPPB and PPT scores, while one study (26) found no significant change. A plausible explanation may be sought in the total duration of training, as a lack of improvement on these tests may stem from shorter duration of an intervention (10 weeks (26) *vs* 15/16 weeks (25, 31)). Moreover, differences in sex distribution could partially explain the dicrepant results. Balachandran (25) and Stoever (31) observed an improvement in SPPB in a sample of both women and men, whereas Vasconcelos et al. (26) found no improvement in a sample composed solely of women. It should be noted, however, that the study population in the Vasconcelos study (26) had started with high baseline SPPB scores, for which no significant additional improvement could be achieved. The study population in the studies by Balachandran (25) and Stoever (31) were composed of both sexes; though the results were not stratified by sex, there was a statistically significant improvement between pre- and post-training. It would be helpful to have data on the the effects of differences exercise (strength, aerobic, power training, etc.) separately for men and women.

### Gait speed test

Gait speed (50), also measured by the 5-meter walk test or the 10-meter walk test, is an easy to administer and reliable tool for assessing physical performance in older adults (31). Three studies (28–30) showed an increase in gait speed; two of them (29, 30) found a marked increase when exercise was combined with elastic resistance training and one (28) reported an increase after electromyostimulation training. The remaining two studies (26, 27) found no change in gait speed. Abellan Van Kan et al. (15) reported that gait speed at usual pace is a consistent risk factor for disability, cognitive impairment, institutionalization, falls, and/or mortality. The authors went on to state that older adults who walk faster than 1.0 m/s generally have a lower risk of disease and a better survival rate. In addition, they suggested a cut off of 0.8 m/s for identifying risk of adverse outcomes when using a 4-m test course and 0.6 m/s as a threshold to predict further functional decline in older adults with impaired mobility. Finally, Peel et al. (51), found gait speed to be an important measure in evaluating comprehensive geriatric syndrome because it is a quick, inexpensive, and reliable measure of functional capacity with a well-documented predictive capability for major health-related outcomes.

A recent review by Hsu et al. (8) reported that gait speed test scores were higher after resistance exercise training than after combined exercise in adults with sarcopenic obesity. The authors reasoned that since obesity reduces the physical capacity of individuals with sarcopenia and since resistance exercise is an optimal way to increase muscle strength, physical performance (such as gait speed) will improve in adults with sarcopenic obesity submitted to a resistance exercise protocol. Considerable improvement in gait speed was noted after administration of a structured and progressive resistance exercise protocol with the use of elastic bands three times a week for a total of 12 sessions, as done in two studies (29, 30). Differently, no significant improvements were observed in the study (27) that employed a bodyweight resistance exercise or in another (26) in which training frequency was twice a week for a total of 10 sessions. A Canadian study (52) involving both men and women reported that the group with sarcopenic obesity and the obese non-sarcopenic group had similar but lower fitness levels (as measured with the gait speed, chair stand, and TUG tests) than the normal weight non-sarcopenic subjects. Obesity rather than sarcopenia seems the predominant factor in reduced physical fitness in such a population. Similarly, a Korean study (53) showed that, although not all results reached statistical significance, the men who engaged in resistance exercise or gait speed training were less likely to develop sarcopenic obesity than those who did not engage in any type of physical exercise. A similar albeit slightly weaker association were found for the women. In their systematic review (54), Graham et al. focused on gait speed and noted that the wide variety of protocols for measuring gait speed left physical performance scores open to different interpretations. Another review (55) reported that gait speed tests may differ according to the pace strategy (normal or maximum speed) and depending whether the subject starts to walk from a static position or when already in motion. Other variables are the distance covered (range, 4 to 500 meters) or the sample population characteristics. As a consequence, the lack of standardization of the walk test protocol can limit comparisons between groups. The response to different types of exercise (strength, aerobics, power training, etc.) as measured on physical performance tests needs to be categorised based on universally agreed cut offs in order to compare different types of populations and training protocols.

### Chair stand test and timed chair rise

Measuring lower body strength is critical for assessing functional performance in older adults. The 30-s chair stand test, also known as the TCR test, is a commonly used tool (56). Three studies showed an improvement on this task with the use of elastic resistance training (29, 30) or traditional resistance training (32). A recent study (57) found the 30-s chair stand test useful for evaluating lower muscle strength in community-dwelling older adults and concluded that by establishing an optimal cut off test could be a useful diagnostic tool for assessing sarcopenia risk in older Japanese participants. Liao et al. (29, 30) found that elastic resistance training led to improvement in muscle mass and physical capacity as measured with the TCR test in a sample of women with sarcopenic obesity. Cesari et al. (58) proposed a cut off ≥17 s on the 5 times sit-to-stand chair stand test in the workup of diagnostic sarcopenic obesity and a range of 15 to 9 s and 17 to 9 s on the 30-s chair stand test for older women and men, respectively (44).

One limitation of some of these studies is that the structural features of the chair (height, length, presence or absence of armrests) are either not reported or that chair height differs, making comparison between studies difficult. One study (59) showed that chair characteristics can affect test performance. For example, a lower seat height may make it more difficult to perform the task, while a higher seat height may decrease the amount of work required at the hip and knee and make the task easier. In addition, a recent study (60) involving a population with sarcopenia used a so-called “sit-to-stand” test that proved useful for measuring physical performance and muscle power in particular. This inexpensive test could yield information on muscle quality and contractile muscle capacity in adults with sarcopenic obesity. It would be helpful to agree on the use of a single test depending on the type of physical exercise carried out and the type of population under study.

### Single leg stance and timed up and go test

Low muscle mass and strength are associated with impaired balance and a concomitant increase in the risk of falls (61). Two studies (29, 30) of the three studies testing balance reported an improvement and both included elastic resistance training, while one (32) reported a decrease in TUG test scores. In their recent review (62), Barri et al. observed that shorter time is a better indicator of functional performance and that a TUG test score ≥13.5 s is a benchmark to identify people at greater risk of falls in a community setting. However, a wide range of cut offs from 10 to 33 seconds are reported in the literature (63). Springer et al. (64) stated that the SLS test is a valid method to quantify static balance ability. They found that the different in timing is not sex-specific but rather age-related, with the eyes open condition resulting in much longer time on the task than the eyes closed condition. In addition, the study set performance criteria based on age group for eyes closed or open in a healthy older population. Nevertheless, there are no studies that provide an indicative cut off for adults with sarcopenic obesity. The role of these tests needs to be explored according to the type of training, with the cut offs adjusted for age and disease in adults with sarcopenic obesity.

### Strengths and limitations

This systematic review has several strengths: the importance of using validated and objective tools to evaluate the effect of training programmes on physical performance in adults with sarcopenic obesity; maintaining or increasing physical function is key to counteract aging-related decline and preserve autonomy; the details of training protocols were reported according to the FITT principle (training frequency, intensity, time, type) which constitute essential features for comparison among studies.

The limitations are: the study authors used heterogeneous criteria for defining sarcopenic obesity and consensus on the definition of sarcopenic obesity is lacking; also lacking is standardization of training program duration (range, 10-26 weeks) and of physical performance in response to the type exercise prescribed (strength, aerobics, power training, etc.); the study samples were composed mainly of women, with moderate to high risk of bias.

### Future directions

The success of a training intervention depends on the barriers that affect an individual’s willingness to engage in physical exercise: personal and health aspects (e.g., physical, and psychological well-being), the surrounding environment, behavioral aspects (e.g., motivation, social support, goal setting, positive affect, self-efficacy), and parameters of physical exercise (e.g., frequency, duration, type, goal attainment, enjoyment) (65). Our review shows poor involvement of older men perhaps because they are less willing than women to participate. A previous study involving men over 65 years found that health aspects and enjoyment are two essential parameters for taking up daily exercise; in contrast, lack of interest/motivation, lack of time, and feeling awkward are common barriers to participation in exercise interventions (66). On this note, administering questionnaires to better understand exercise modality preferences and to maximize adherence to prescription in this population and in men especially is warranted. Other promising approaches to improve engagement in long-term exercise might be: i) to increase awareness about the beneficial effects of exercise; ii) to set achievable and measurable goals in an enjoyable and sociable environment.

Standardization of training and common guidelines designed on the FITT principle for enhancing physical performance is within reach. However, the difference in type of exercise (physical capacity and strength components to train, intensity, and volume), spaces, and exercise equipment, time availability (number of sessions and duration per week) make standardization problematic. Given these circumstances, collaboration between clinicians and kinesiologists is fundamental for the design, evaluation, and prescription of tailored exercise training programs.

Despite the lack of a standardization in the sarcopenia screening, future works should include all the most current and updated definitions (e.g., the European (9), the Asian (11), the International (10) working groups and the ESPEN and EASO groups (17)) in order to have a clear picture of the best approach useful in identifying the prevalence of sarcopenia in older adults with obesity.

## Conclusion

Physical capacity decreases with age and the decline is steeper in sedentary adults with sarcopenic obesity. Physical exercise, and progressive resistance training in particular, is the most used training modality in adults aged 60-80 years. Of note, none of the previous trials explored differences in exercise prescription by classifying participants in subgroups based on age-level. This is a key aspect to develop in the future.

Outcomes show that physical performance is improved or at least maintained as assessed with SPPB, PPT, Gait Speed, TCR, Chair Stand, and SLS tests. Nonetheless, whether better results can be achieved with other types of training remains to be elucidated. It follows then that other types of functional tests for evaluating muscle function (i.e., muscle mechanical power) should be applied.

Although most of the studies only involved women, the study sample should include more older men in order to comprehensively investigate different types of training (aerobic, power training, combination of these modalities) and better understand whether different protocols could yield greater and faster benefits for physical performance outcomes. In addition, the most recent definition for sarcopenia screening should be considered. Finally, interventions of longer duration with follow-up assessment after the training period could demonstrate the actual effectiveness of exercise in improving physical function in adults with sarcopenic obesity.

## Data availability statement

The raw data supporting the conclusions of this article will be made available by the authors, without undue reservation.

## Author contributions

LG reviewed the literature, collected the data, and drafted the manuscript, VM, TT, and AR revised and edited the manuscripts critically, VM, TT, FS, and AR supervised the manuscript. All authors contributed to the article and approved the submitted version.

## Acknowledgements

The authors wish to thank Kenneth Adolf Britsch for his assistance in reading the manuscript.

## Conflict of interest

The authors declare that the research was conducted in the absence of any commercial or financial relationships that could be construed as a potential conflict of interest.

## Publisher’s note

All claims expressed in this article are solely those of the authors and do not necessarily represent those of their affiliated organizations, or those of the publisher, the editors and the reviewers. Any product that may be evaluated in this article, or claim that may be made by its manufacturer, is not guaranteed or endorsed by the publisher.

## Abbreviations

Physical Performance Test (PPT), Short Physical Performance Battery (SPPB), Single Leg-Stance (SLS), Time Up & Go test (TUG), Timed chair rise (TCR).
